# Prevalence and factors associated with undiagnosed and uncontrolled heart disease: A study based on self-reported chronic heart disease and symptom-based angina pectoris among middle-aged and older Indian adults

**DOI:** 10.1371/journal.pone.0287455

**Published:** 2023-06-28

**Authors:** Waquar Ahmed, T. Muhammad, Chanda Maurya, Saddaf Naaz Akhtar

**Affiliations:** 1 Tata Institute of Social Sciences, School of Health Systems Studies, Mumbai, India; 2 Department of Family & Generations, International Institute for Population Sciences, Mumbai, India; 3 Department of Survey Research and Data Analytics, International Institute for Population Sciences, Mumbai, India; 4 Faculty of Social, Human and Mathematical Sciences, Centre for Research on Ageing, University of Southampton, Southampton, United Kingdom; Bangladesh University of Health Sciences, BANGLADESH

## Abstract

**Background:**

This study aimed to examine the prevalence of heart diseases and angina pectoris and associated factors among middle-aged and older Indian adults. Additionally, the study examined the prevalence and associated factors of undiagnosed and uncontrolled heart disease among middle-aged and older adults based on self-reported chronic heart disease (CHD) and symptom-based angina pectoris (AP).

**Methods:**

We used cross-sectional data from the first wave of the Longitudinal Ageing Study of India, 2017–18. The sample consists of 59,854 individuals (27, 769 males and 32,085 females) aged 45 years and above. Maximum likelihood binary logistic regression models were employed to examine the associations between morbidities, other covariates (demographic factors, socio-economic factors and behavioral factors) and heart disease and angina.

**Results:**

A proportion of 4.16% older males and 3.55% older females reported the diagnosis of heart diseases. A proportion of 4.69% older males and 7.02% older females had symptom-based angina. The odds of having heart disease were higher among those who were hypertensive and who had family history of heart disease, and it was higher among those whose cholesterol levels were higher. Individuals with hypertension, diabetes, high cholesterol and family history of heart disease were more likely to have angina than their healthy counterparts. The odds of undiagnosed heart disease were lower but the odds of uncontrolled heart disease were higher among those who were hypertensive than non-hypertensive individuals. Those having diabetes were less likely to have undiagnosed heart disease while among the diabetic people the odds of uncontrolled heart disease were higher. Similarly, higher odds were observed among people with high cholesterol, having stroke and also among those who had a history of heart disease than their counterparts.

**Conclusions:**

The present study provided a comparative prevalence of heart disease and agina and their associations with chronic diseases among middle-aged and older adults in India. The higher prevalence of undiagnosed and uncontrolled heart disease and their risk factors among middle-aged and older Indians manisfest alarming public health concerns and future health demand.

## Background

Globally, cardiovascular diseases (CVDs) are the leading cause of death. According to estimates, 17.9 million deaths worldwide in 2019 were attributable to CVDs. Heart attack and stroke deaths accounted for 85% of these death. In 2019, non-communicable diseases accounted for 17 million premature deaths under the age of 70, of which 38% were due to CVDs [1]. Total CVD cases nearly doubled from 271 million in 1990 to 523 million in 2019, and CVD-related death increased from 12.1 million in 1990 to 18.6 million in 2019 [2]. Annually, there are more than 10.5 million deaths in India, and it was also reported that 20.3% of the deaths in males and 16.9% of the deaths in women were attributed to CVD [3]. Early detection of cardiovascular illness is crucial for effective treatment with counselling and medication [1].

Angina pectoris (AP), is a common symptom of coronary artery disease (CAD) with myocardial hypoxia resulting from either obstructive or non-obstructive CAD that can have an adverse effect on a person’s quality of life [4,5]. It can also result from non-CAD conditions, such as anaemia, hyperthyroidism, respiratory illnesses, and valvular disease [4,6]. The principal cause of angina and myocardial infarction continues to be atherosclerosis of the large coronary arteries. Angina, the chest pain that develops when pain-inducing chemicals build up in the myocardium, is caused by atherosclerotic constriction in coronary arteries that reduces the lumen of a coronary artery by more than 75%. [7]. It is possible that different genetic pathways may be implicated in the diverse coronary heart disease (CHD) phenotypes since the functional characteristics of the coronaries may vary between CHD individuals with or without angina [8]. According to the SCOT HEART trial, patients with stable chest pain are at risk for having adverse cardiac events [9].

Early CHD detection lowers the risk of future major illness and is crucial for early interventions. The Rose Angina Questionnaire (RAQ), a standardised tool for quantifying typical angina in population surveys, is one of the simple and affordable techniques used in population-based studies to assess high risk individuals [10,11]. The diagnosis of angina is clinical and based on a thorough history of the characteristics of the pain, with assessment of its quality, location, radiation, frequency, severity, duration, and concomitant symptoms as well as to assess triggering and mitigating factors [11,12].

In order to identify ischemic heart pain (angina pectoris and myocardial infarction) for epidemiological surveys, the RAQ was developed in 1962 [11]. Since that time, epidemiological research has employed the RAQ in many nations to identify CHD. Chest pain has historically been ascribed to ischemia. However, more recent data indicate that 70–80% of ischemia episodes in people with coronary artery disease may really be asymptomatic [7]. Accelerated atherosclerosis results in ischemic heart disease with angina pectoris and myocardial infarctions [7]. It was primarily evaluated as a predictor of coronary morbidity and mortality [13–15]. The RAQ was initially created using responses from men alone [11]. Since then, a number of studies have been undertaken with participants from both sexes [16,17], and have suggested that the RAQ is suitable to use for women as well.

Rose reported that the questionnaire’s sensitivity and specificity might differ between nations [18]. Numerous research have been done since then to validate the RAQ. In large-scale epidemiological surveys, the RAQ, which has a moderate sensitivity but high specificity to detect CHD, can be used to evaluate individual at risk [19]. The questionnaire showed a sensitivity of 53–86% and a specificity of 70–89% in comparison to clinical diagnoses, exercise echocardiograms (ECGs), and myocardial perfusion scans [19,20]. In addition to this, another study also reported varying sensitivity and specificity of the RAQ depending on the gold standard employed, but generally, there has been high specificity (80–95%) but variable sensitivity (19–83%) [21].

One study reported that the Rose questionnaire, which is a reliable screening tool, is suitable to estimate the risk and provide details on undiagnosed CHD in both sexes [10]. Angina may be undiagnosed in individuals without established CVD for a variety of reasons [22]. Mansournia et al., also reported that each RAQ and ECG plays a unique role in predicting CHD occurrences. Even in the presence of a normal ECG, the Rose questionnaire can be used as a quick and effective clinical screening tool in Iranians who have a high prevalence of CHD [10]. Similarly one previous study reported that according to the RAQ, self-report, and ECG in Tehran city, the prevalence of CHD was 10.7%, 6%, and 11.8%, respectively [23]. Moreover, one study reported a statistically significant relationship between AP and measures of psychological distress. Additionally, they found that AP was independently associated with a history of heart illness and gastrointestinal medications [6]. McKee et al., reported that undiagnosed angina is associated with low educational attainment [24]. Furthermore, men are more likely than women to suffer from angina. Patients who are socioeconomically disadvantaged have a higher likelihood of having angina yet are less likely to visit their general practitioner [25].

It is well documented that the chronic conditions in low and middle income countries are often under-reported and undiagnosed and as a result, they are untreated which increase the risk of multi-morbidity and disability burden [26–28]. Understanding the factors that hinder the diagnosis and control of heart disease will help frame policies and interventions that can effectively target the vulnerable populations. This study aimed to examine the prevalence of heart diseases and angina and associated factors among Indian adults. Additionally, the study examined the prevalence and associated factors of undiagnosed and uncontrolled heart disease among middle-aged and older adults based on self-reported CHD and symptom-based AP.

## Data and methods

### Data

We used data from the Longitudinal Ageing Study of India (Wave-1), 2017–18, a cross-sectional large-scale sample survey that provides reliable, nationally representative statistics on the ageing and health. The survey has been conducted under the Ministry of Health and Family Welfare (MoHFW), Government of India. International Institute for Population Sciences (IIPS) collaborated with Harvard T. H. Chan School of Public Health and the University of Southern California to implement the survey. The survey employed a multistage stratified area probability cluster sampling design and interviewed a total of 73, 396 individuals. LASI data set provides information on individuals aged 45 and above and their spouse who resides in the same household. It provides information from personal individual with standardized physical examination, symptoms based assessments of the health conditions and laboratories tests. The data are available at The Gateway to Global Aging Data (https://g2aging.org/). The LASI dataset used in the present study includes 59,854 individuals (27, 769 males and 32,085 females) aged 45 years and above, who participated in the health survey and physical examinations. The final data set for multivariable analysis of undiagnosed and uncontrolled heart disease consisted of 5,345 individuals with self-reported heart disease.

### Measures

Angina, a risk factor for ischemic heart disease, refers to chest pain or discomfort, which occurs when the area surrounding the heart muscles does not receive oxygen sufficiently. This arterial blockage interrupts or stops the blood flow to the heart muscles, eventually resulting in the occurrence of angina. The World Health Organization’s Rose Angina Questionnaire [11] was used for the symptom based assessment of angina pectoris. The Rose questionnaire is a screening tool that has been shown to be predictive of ischemic heart disease. Algorithm for the angina consists of 7 questions. Those who reported positive response for all the 7 questions or symptom are considered as having symptoms of angina. During the interview, self-reported heart disease, treatment seeking behavior, family history of heart disease were asked. Individuals who responded affirmatively to the questions of the self-reported heart disease were considered as having heart disease.

### Undiagnosed heart disease

Individuals who responded negatively to the question, “Have any health professionals ever diagnosed chronic heart diseases such as coronary heart disease (heart attack or Myocardial Infarction), congestive heart failure, or other chronic heart problems” but their symptom-based measures showed the presence of angina were considered as having the undiagnosed condition of heart diseases.

### Uncontrolled heart disease

If the individual reported to have been diagnosed with heart disease by a health professional and currently taking treatment and symptom-based measures of angina were positive, they were considered as having uncontrolled heart disease.

### Explanatory variables

We determine the presence of chronic diseases such as hypertension, diabetes, high cholesterol and stroke based on the positive response to the question “have any health professionals ever diagnosed hypertension, diabetes, high cholesterol and stroke” separately. Family history of heart disease was categorized as no and yes.

Additionally, a set of covariates pertaining to three domains were also included in the analysis. These domains were demographic factors, socio-economic factors and behavioral factors. The demographic factors included: age (categorized as 45–59 years, 60–69 years and 70 years or above), sex (male and female), marital status (In-union and not in union), caste (Others, OBC and SC/ST), religion (Hindu and non-Hindu), and place of residence (urban and rural).

The socio-economic factors included education (categorized as illiterate, less than 10 years of education and 10 or more years of education), wealth index (poor, middle and rich). Behavioral factors included body mass index (under-weight, normal-weight, over-weight and obese), smoking (no and yes), use smokeless tobacco (no and yes), drink alcohol (no and yes), vigorous physical activity (no and yes).

### Statistical analysis

To understand the distribution of the study population, descriptive statistics were presented by background characteristics. To estimate the differentials of the heart disease and angina, bi-variate percentage distribution was calculated. The results were tested for statistically significant independence using Pearson’s Chi-squared test statistic. Further, to find out the association between morbidities, other covariates (demographic factors, socio-economic factors and behavioral factors) and heart disease and angina, maximum likelihood binary logistic regression methods were employed. The results are presented as adjusted odds ratios (OR) with 95% confidence intervals (CI).

Further bi-variate percentage distribution was calculated to find out the differentials of the undiagnosed and uncontrolled heart diseases and examined the statistical significance using Pearson’s chi-squared statistics. To show the association between morbidities, other covariates (demographic factors, socio-economic factors and behavioral factors) and undiagnosed and uncontrolled heart diseases, maximum likelihood binary logistic regression models were used and the results are presented as adjusted OR with 95% CIs.

## Results

**Fig 1:**
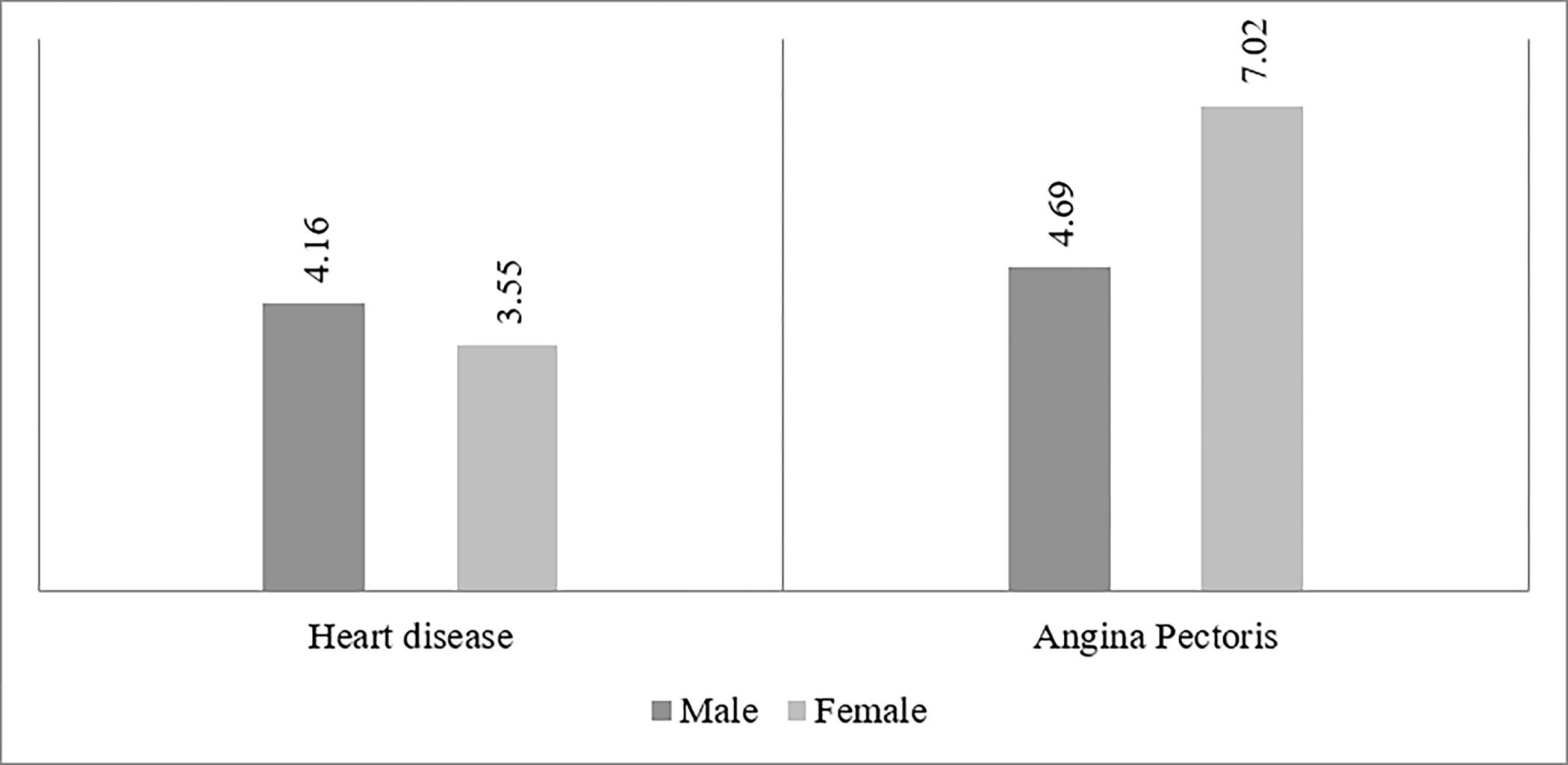
Prevalence of self-reported heart disease and symptoms based angina pectoris among older adults. Note: Chi-Square test showed that the difference between male and female was significant at p<0.001 for both heart disease and angina pectoris.

**Table 1** indicates the demographic characteristics for all the background variables. About half of the individuals belonged to 45–59 age-group. Nearly 54% of the sample population was female, and 46% was male. About 51% individuals were illiterate, 42% were from the poor wealth quintiles, and 66% people resided in the rural areas. Approximately 29% of the sample population was hypertensive, 13% individuals were diabetes, 4% people had high cholesterol, and 1.66% people suffered from stroke. Fig 1 shows the prevalence of heart disease and angina among older adults. Nearly 4.16% of males and 3.55% females were diagnosed with heart disease, whereas, 4.69% of males and 7.02% of females had symptoms of angina.

**Table 1:** Sample charactistics of study population and prevalence of heart disease by background charactistics.

Background variables	Study population	
	N	%
**Age group (in years)**		
45–59	31,486	50.07
60–69	17,411	30.01
70+	10,957	19.92
**Sex**		
Male	27,769	46.39
Female	32,085	53.61
**Marital status**		
In union	44843	73.85
Not in union	15,010	26.15
**Education**		
No	28,162	50.68
less than 10 years	20674	31.85
10 or more years	11,018	17.47
**Wealth**		
Poor	23,912	42.34
Middle	12,094	20.39
Rich	23848	37.26
**Caste**		
others	16,485	27.54
OBC	22,704	37.93
SC/ST	20,665	34.53
**Religion**		
Hindu	43,772	82.51
Non-Hindu	16,082	17.49
**Residence**		
Rural	39,314	65.68
Urban	20,540	34.32
**Hypertension**		
Yes	17,177	28.71
No	42,658	71.29
**Diabetes**		
Yes	7,635	12.76
No	52,199	87.24
**High cholesterol**		
Yes	2,096	3.51
No	57,746	96.50
**Stroke**		
Yes	927	1.66
No	58,927	98.34
**Family history of heart disease**		
Yes	4,559	8.00
No	55,096	92.00
**BMI**		
Underweight	10,992	18.36
Normal weight	31,311	52.31
Overweight	13,119	21.92
Obese	4,432	7.41
**Smoking**		
No	51,635	86.27
Yes	8,219	13.73
**Smokeless tobacco**		
No	48,273	80.65
Yes	11,581	19.35
**Alcohol**		
No	58,592	97.89
Regular	1,262	2.11
**Physical activity**		
No	35,918	60.01
Yes	23,936	39.99

Notes: OBC: Other backward classes; SC/ST: Scheduled caste/ scheduled tribe; BMI: Body mass index.

**Fig 1** presents the prevalence of self-reported heart disease and symptoms based angina among older adults. A proportion of 4.16% older males and 3.55% older females reported the diagnosis of heart disease. A proportion of 4.69% older males and 7.02% older females had symptom-based angina.

**Table 2** represents the prevalence and logistic regression estimates of heart disease and angina. The prevalence of heart disease (6.48%) and angina (6.13%) were significantly higher among those age 70 years and above. The prevalence of angina was higher among females while heart disease was more prevalent among males. Education showed a significant relationship with heart disease and angina, and the prevalence of heart disease was reported among those who were highly educated (10 years and above) (6.23%) but angina was more prevalent among illiterate people (6.85). In addition, morbidities showed significant and positive association with both heart disease and angina. Heart disease was more prevalent among individuals having hypertension, diabetes, high cholesterol and stroke. The prevalence of heart disease was higher among those who had family history of heart disease (7.68%) and those who were obese (9.66%).

**Table 2:** Prevalence and regression estimates of heart disease and angina pectoris by background charactistics.

Background variables	Heart disease				Angina pectoris			
	N	%	P-value	OR 95% (CI)	N	%	P-value	OR 95% (CI)
**Age group (in years)**			<0.001				0.001	
45–59	740	2.40		Ref.	1,736	5.67		Ref.
60–69	799	4.46		1.67***(1.5 1.86)	1,087	6.00		1.08*(0.99 1.17)
70+	625	6.48		2.03***(1.79 2.29)	673	6.13		1.06 (0.96 1.18)
**Sex**			<0.001				<0.001	
Male	1,165	4.00		Ref.	1,259	5.00		Ref.
Female	999	3.55		0.61***(0.54 0.68)	2,237	7.02		1.54***(1.41 1.69)
**Marital status**			0.750				0.007	
In union	1,615	3.65		Ref.	2,552	5.01		Ref.
Not in union	549	4.00		0.91 (0.81 1.02)	944	6.00		0.91**(0.83 0.99)
**Education**			<0.001				<0.001	
No	739	2.77		Ref.	1,905	6.85		Ref.
Less than 10 years	859	4.20		1.11*(0.99 1.24)	1,195	5.98		0.93 (0.86 1.01)
10 or more years	566	6.23		0.92 (0.8 1.06)	396	3.30		0.61***(0.53 0.69)
**Wealth**			<0.001				0.003	
Poor	631	2.79		Ref.	1,315	5.74		Ref.
Middle	416	3.42		1.16**(1.02 1.32)	698	6.00		1.04 (0.94 1.14)
Rich	1,117	5.24		1.34***(1.2 1.49)	1,483	6.11		1.11**(1.03 1.21)
**Caste**			<0.001				0.501	
Others	866	4.92		Ref.	993	6.22		Ref.
OBC	829	3.90		0.9*(0.81 1)	1,308	5.74		0.91**(0.84 1)
SC/ST	469	2.70		0.7***(0.62 0.79)	1,195	6.00		0.9**(0.82 0.99)
**Religion**			<0.001				0.917	
Hindu	1,490	3.63		Ref.	2,554	5.76		Ref.
Non-Hindu	675	4.77		1.06 (0.95 1.17)	942	6.87		0.97 (0.9 1.05)
**Residence**			<0.001				<0.001	
Rural	1,086	3.00		Ref.	2,596	6.70		Ref.
Urban	1,078	6.00		1.27***(1.15 1.4)	900	4.22		0.66***(0.6 0.72)
**Hypertension**			<0.001				<0.001	
No	786	1.82		Ref.	2,118	7.79		Ref.
Yes	1,378	9.00		2.88***(2.6 3.18)	1,377	5.27		1.67***(1.55 1.81)
**Diabetes**			<0.001				0.001	
No	1,484	3.00		Ref.	2,986	6.38		Ref.
Yes	680	10.65		1.47***(1.32 1.63)	509	5.90		1.1*(0.99 1.23)
**High cholesterol**			<0.001				<0.001	
No	1,783	4.00		Ref.	3,312	8.58		Ref.
Yes	380	16.8		3.08***(2.69 3.53)	184	5.89		1.45***(1.23 1.71)
**Stroke**			<0.001				0.025	
No	2,041	3.66		Ref.	70	8.20		Ref.
Yes	123	14.08		1.9***(1.54 2.34)	3,426	5.92		1.16 (0.9 1.49)
**Family history of heart disease**			<0.001				<0.001	
No	1,735	3.52		Ref.	329	6.84		Ref.
Yes	424	7.68		2.36***(2.1 2.66)	3,146	5.90		1.29***(1.14 1.46)
**BMI**			<0.001				<0.001	
Underweight	215	2.09		Ref.	706	6.45		Ref.
Normal weight	1,058	4.00		1.28***(1.1 1.5)	1,751	5.88		0.9**(0.82 0.98)
overweight	642	4.35		1.33***(1.12 1.58)	740	5.60		0.89**(0.79 1)
obese	249	9.66		1.28**(1.04 1.58)	299	6.01		1.01 (0.86 1.17)
**Smoking**			<0.001				0.544	
No	1,915	4.00		Ref.	3,004	6.00		Ref.
Yes	249	3.17		0.95 (0.81 1.1)	492	5.68		1.24***(1.11 1.38)
**Smokeless tobacco**			0.002				0.844	
No	1,869	4.14		Ref.	2,815	5.91		Ref.
Yes	295	2.68		0.85**(0.74 0.97)	681	6.13		1.08*(0.99 1.19)
**Alcohol**			0.042				<0.001	
No	1,811	3.94		Ref.	2,964	6.18		Ref.
Yes	353	3.25		0.89*(0.78 1.02)	532	4.68		0.95 (0.85 1.06)
**Physical activity**			<0.001				0.228	
No	1,591	4.86		Ref.	2,064	5.80		Ref.
Yes	573	2.00		0.73***(0.66 0.81)	1,432	6.18		1.15***(1.07 1.24)
**Constant**				0.01***(0.01 0.02)				0.05***(0.04 0.06)

Notes: OBC: Other backward classes; SC/ST: Scheduled caste/ scheduled tribe; BMI: Body mass index.

The odds of heart disease were 2 times higher (OR: 2.03, CI: 1.79–2.29) among those aged 70 years and above. Females showed lower odds of having heart disease (OR: 2.03, CI: 1.79–2.29), whereas showed higher odds of angina (OR: 2.03, CI: 1.79–2.29) than males. Individuals with 10 or more years of education were 39% less likely to have angina (OR: 0.61, CI: 0.53–0.69) than illiterate individuals. Individuals from the rich wealth quintiles were more likely to have both heart disease and angina compared to those from lower wealth quintiles. The odds of having heart disease were 2 times higher among those who were hypertensive (OR: 2.88, CI: 2.6–0.3.18) and who had family history of heart disease (OR: 2.36, CI: 2.1–2.66), whereas, it was 3 times higher among those whose cholesterol level was higher (OR: 3.08, CI: 2.69–3.53). Individuals with hypertension (OR: 1.67, CI: 1.55–1.81), diabetes (OR: 1.10, CI: 0.99–1.23), high cholesterol (OR: 1.45, CI: 1.23–1.71) and family history of heart disease (OR: 1.29, CI: 1.14–1.46) were 67%, 10%, 45% and 29% more likely to have angina than their healthy counterparts. The odds of having angina were higher among those who smoked and those who used smokeless tobacco as compared to those who did not smoke or use smokeless tobacco.

**Fig 2:**
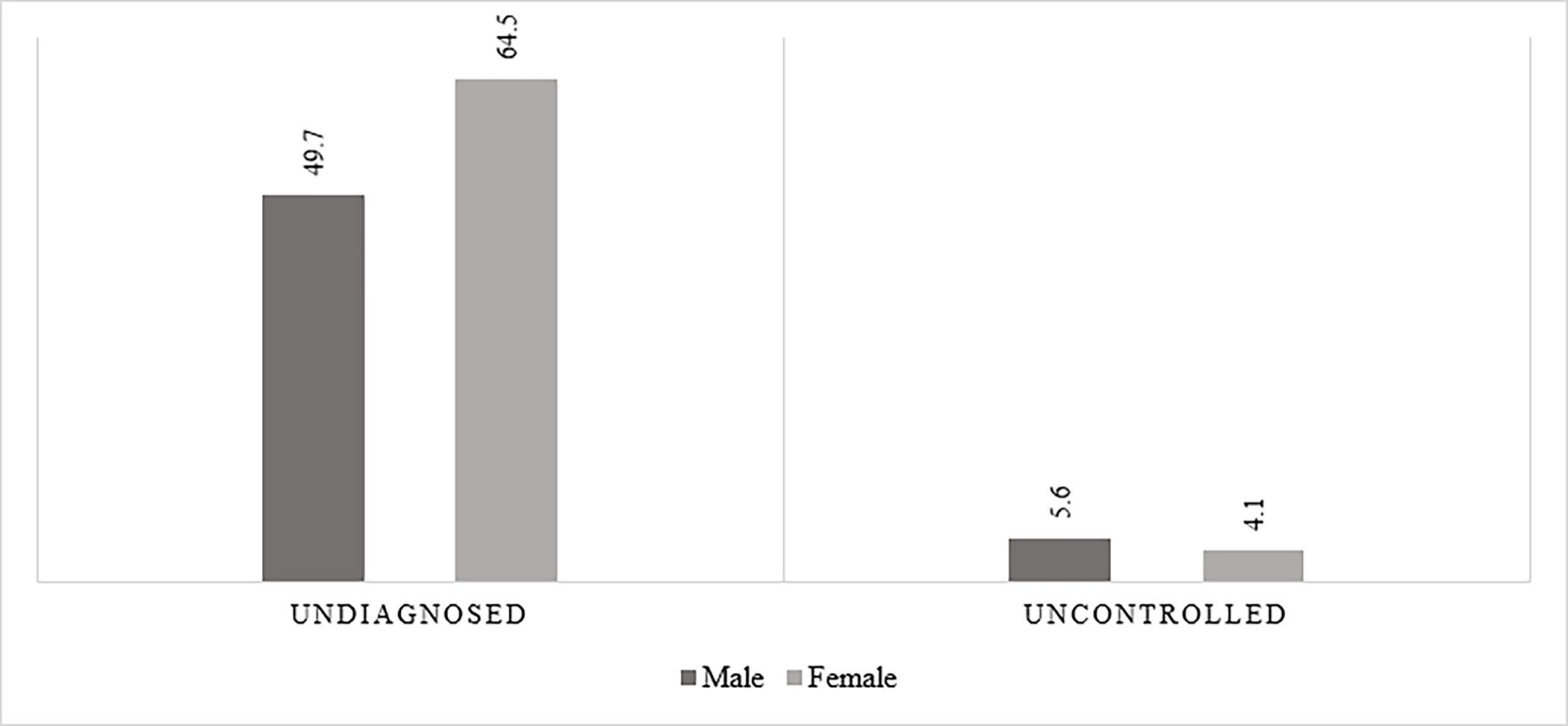
Prevalence of undiagnosed and uncontrolled heart diseases among older adults. Note: Chi-Square test showed that the difference between male and female was significant at p<0.001 for both undiagnosed and uncontrolled heart diseases.

**Fig 2** presents the prevalence of undiagnosed and uncontrolled heart disease among older adults. A proportion of 4.16% older males and 3.55% older females reported the diagnosis of heart diseases. The prevalence of undiagnosed heart disease was 64.5% among females and 49.7% among male older adults. Out of those having heart disease and taking any treatment or mediacation, 5.6% female and 4.1% male having uncontrolled heart dieases.

**Table 3** represents the prevalence and regression analysis estimates of undiagnosed and uncontrolled heart disease among older adults by their background characteristics. The prevalence of undiagnosed heart disease (68.69%) was higher among adults aged 45–49 years, while uncontrolled heart disease (5.95%) was more prevalent among older adults aged 70 or more years. 64.5% of females were having undiagnosed heart disease, while the prevalence of uncontrolled heart disease was higher among males. Among individuals who belonged to poor wealth quintiles, 65.21% had undiagnosed heart disease but the prevalence of uncontrolled heart disease was higher among richer people. The prevalence of undiagnosed heart disease was lower among those having hypertension, diabetes and high cholesterol, while those who were having hypertension, diabetes and high cholesterol had higher prevalence of uncontrolled heart disease. The prevalence of undiagnosed heart disease was higher among those who smoked (61.9%) or used smokeless tobacco (68.04%). The odds of having undiagnosed heart disease were less likely among those aged 60–69 (OR: 0.68, CI: 0.58–0.78) and 70 or more years (OR: 0.59, CI: 0.49–0.71) than adults aged 45–59 years.

**Table 3:** Prevalence and regression estimates of undiagnosed and uncontrolled heart disease by background charactistics.

Background variables	Undiagnosed				Uncontrolled			
	N	%	P-value	OR 95% (CI)	N	%	P-value	OR 95% (CI)
**Age group (in years)**			<0.001				0.001	
45–59	1,622	68.69		Ref.	81	3.69		Ref.
60–69	980	55.92		0.68***(0.58 0.78)	84	4.99		1.14 (0.83 1.58)
70+	580	44.71		0.59***(0.49 0.71)	74	5.95		1.29 (0.9 1.85)
**Sex**			<0.001					
Male	1,105	49.68		Ref.	125	5.57	0.002	Ref.
Female	2,077	64.50		2.74***(2.33 3.23)	114	4.08		0.61***(0.43 0.85)
**Marital status**			0.231					
In union	2,328	59.69		Ref.	171	4.47	0.427	Ref.
Not in union	854	55.01		0.92 (0.79 1.08)	68	5.27		1.25 (0.9 1.73)
**Education**			<0.001					
No	1,796	69.67		Ref.	81	4.05	<0.001	Ref.
Less than 10 years	1,058	55.81		0.79***(0.68 0.92)	105	5.21		1.32*(0.96 1.82)
10 or more years	328	29.80		0.66***(0.54 0.81)	53	5.58		1 (0.65 1.53)
**Wealth**			<0.001					
Poor	1,228	65.21		Ref.	53	4.43	<0.001	Ref.
Middle	630	62.12		0.86*(0.72 1.03)	56	4.27		1.81***(1.23 2.68)
Rich	1,324	50.85		0.78***(0.67 0.9)	130	5.10		1.7***(1.21 2.4)
**Caste**			<0.001					
Others	878	52.70		Ref.	91	4.96	0.089	Ref.
OBC	1,181	57.09		1.08 (0.92 1.25)	90	4.56		1.03 (0.75 1.41)
SC/ST	1,123	67.45		1.39***(1.17 1.64)	58	4.61		1.04 (0.73 1.5)
**Religion**			<0.001					
Hindu	2,340	59.01		Ref.	157	4.48	0.037	Ref.
Non-Hindu	842	56.04		0.87*(0.75 1)	82	5.49		1.16 (0.87 1.55)
**Residence**			<0.001					
Rural	2,423	67.31		Ref.	125	3.85	<0.001	Ref.
Urban	759	37.72		0.53***(0.46 0.61)	114	6.64		1.27 (0.94 1.71)
**Hypertension**			<0.001				<0.001	
No	2,008	73.17		Ref.	80	3.04		Ref.
Yes	1,173	41.16		0.51***(0.45 0.58)	159	6.61		1.54***(1.14 2.09)
**Diabetes**			<0.001				<0.001	
No	2,782	65.25		Ref.	80	3.68		Ref.
Yes	399	30.86		0.66***(0.56 0.77)	159	8.74		1.6***(1.18 2.16)
**High cholesterol**			<0.001				<0.001	
No	3,057	60.38		Ref.	188	4.34		Ref.
Yes	125	25.13		0.43***(0.34 0.54)	51	10.60		1.62***(1.13 2.32)
**Stroke**			<0.001				<0.001	
No	54	29.41		Ref.	13	10.24		Ref.
Yes	3,128	59.48		0.55***(0.38 0.79)	226	4.48		1.06 (0.58 1.93)
**Family history** **of heart disease**			<0.001				<0.001	
No	266	41.08		Ref.	50	8.60		Ref.
Yes	2,916	60.59		0.5***(0.42 0.61)	189	4.20		1.46**(1.04 2.05)
**BMI**			<0.001					
Under weight	665	74.6		Ref.	22	1.48	0.022	Ref.
Normal weight	1,590	59.26		0.72***(0.6 0.88)	127	6.23		1.38 (0.86 2.23)
Over weight	665	53.90		0.69***(0.55 0.86)	65	4.68		1.14 (0.67 1.93)
obese	262	34.82		0.79*(0.6 1.04)	25	3.38		1.03 (0.55 1.94)
**Smoking**			0.003					
No	2,728	57.91		Ref.	209	4.70	0.780	Ref.
Yes	454	61.90		1.28**(1.04 1.56)	30	4.65		1.02 (0.66 1.57)
**Smokeless tobacco**			<0.001					
No	2,542	56.10		Ref.	214	5.15	0.003	Ref.
Yes	640	68.04		1.24**(1.05 1.47)	25	2.76		0.67*(0.43 1.03)
**Drink alcohol**			0.305					
No	2,696	58.71		Ref.	204	4.72	0.648	Ref.
Yes	486	56.14		1.19*(0.98 1.45)	35	4.46		0.77 (0.51 1.16)
**Physical activity**			<0.001					
No	1,838	51.11		Ref.	175	5.68	0.003	Ref.
Yes	1,344	70.71		1.62***(1.41 1.87)	64	3.02		0.8 (0.59 1.1)
**Constant**				3.28***(2.44 4.41)				0.02***(0.01 0.03)

Notes: OBC: Other backward classes; SC/ST: Scheduled caste/ scheduled tribe; BMI: Body mass index.

Females were more likely to have undiagnosed heart disease (OR: 2.74, CI: 2.33–3.23) but the odds were lower for the uncontrolled (OR: 0.61, CI: 0.43–0.85) condition than males. The odds of having undiagnosed heart disease were less likely among individuals with less than 10 years and having 10 or more years of education than illiterate individuals. The odds of uncontrolled heart disease were higher among those having less than 10 years of education. Individuals from the middle (OR: 0.86, CI: 0.72–1.03) and rich (OR: 0.78, CI: 0.67–0.90) wealth quintiles were less likely to have undiagnosed heart disease, while individuals from these wealth quintiles were more likely to have uncontrolled heart disease. The odds of undiagnosed heart disease were less likely (OR: 0.51, CI: 0.45–0.58), but the odds of uncontrolled heart disease were more likely (OR: 1.54, CI: 1.14–2.09) among those who were hypertensive than non-hypertensive individuals. Those having diabetes were less likely (OR: 0.66, CI: 0.56–0.77) to have undiagnosed heart disease, while among diabetic people, the odds of uncontrolled heart disease were higher (OR: 1.6, CI: 1.18–2.16). Similar odds were found among people with high cholesterol, having stroke and also among those who had a history of heart disease than their counterparts. Individuals who smoked or used smokeless tobacco were more likely to have undiagnosed heart disease than those who did not smoke or use smokeless tobacco.

## Discussion

### Factors associated with heart disease and angina pectoris

According to the WHO Report 2021, the majority of CVD-related deaths occur in low- and middle-income countries. (LMIC) [29]. Though, there are variety of underlying causes of CVDs which are manifestations of the fundamental forces driving social, economic, and cultural change: globalization, urbanization, and population aging [29]. Despite the prevalence of heart disease being extensively studied, the present study is the first to demonstrate comparative research that looked at the prevalence of heart disease and angina together among older adults in India. The present study assessed the prevalence of heart disease and angina and their interactions with chronic diseases among older Indian adults using LASI-datasets. The findings revealed that the prevalence of heart disease (6.48%) and angina (6.13%) were significantly higher among those age 70 years and above. However, a recent study showed that multimorbidity was highest in chronic heart disease (91%) and lowest in angina (64.8%) in India [30].

Previous studies suggest that the prevalence of CVD increases with increasing age [31], and several CVD risk factors are acquired incrementally during an individual’s lifetime as they age while the prevalence angina rises steeply with increasing age [32]. Our study revealed that older adults aged 70 years and above have two-times greater risk of heart disease and older adults age 60–69 years have higher risk of angina. However, earlier study demonstrates that higher risk of angina pictoris is found among women than men in all age groups [32] and our study has showed the similar results. While previous study argued that women have greater risk of CVD than men among older adults [31] but our findings revealed the contradictory results. Despite the fact that, it is estimated that about 35% of heart attacks in women go unrecognized or unreported.

According to the Global Burden of Disease, CVDs account for approximately one-quarter (24.8%) of all fatalities in India [2]. Poverty, stress, and hereditary characteristics are also CVD risk factors [29]. However, our study showed that individuals from the rich wealth quintiles are significantly more likely to have both heart disease and angina compared to those from lower wealth quintiles. Meanwhile, recent study conducted in Sweden demonstrated that low educational level, unemployment, poor economic situation, indications of depression, and a high level of general stress were all connected with Rose angina [33]. Consistently, our findings revealed that Individuals with higher levels of education were less likely to have angina than uneducated individuals. Furthermore, the present finding revealed that the risk of heart disease were two times higher among those who had family history of heart disease and the WHO factsheet reported that hereditary characteristics are also CVD risk factors [29]. On the other hand, the odds of having heart disease were threefold greater among those cholesterol levels were higher, and similar findings were also depicted in the other study [31]. It is anticipated that 17.6 percent of hypertensive patients worldwide live in India, implying a significant increase in cardiovascular disease burden in the near future [34]. This necessitates early identification and treatment since proper blood pressure control can prevent about one-third of all cardiovascular-related deaths.

Meanwhile, the association between angina and hypertensive heart disease is widely known [35,36]. The most prevalent cause of angina in hypertensive people is coronary artery disease [36]. However, it is conceivable that variables such as left ventricular hypertrophy play a crucial role in hypertensive heart disease patients [37]. Earlier study reported that silent angina has occurred in 10–15% of patients with coronary heart diseases [38]. And the present study revealed that individuals with hypertension were 67% more likely to have angina than their healthy counterparts. A recent scientific report found that the higher cholesterol-to-HDL-C ratio, diabetes, and family history were significantly associated with the occurrence of angina [39–42], and the present finding showed that individuals with high-cholesterol, diabetes and family history of heart diseases were 45%, 10% and 29%, more likely to have angina. Furthermore, previous study suggests that smoking is widespread in individuals with acute myocardial infarction (AMI) and is a significant modifiable risk factor for recurrent episodes [43]. This previous study depicted a gradient in the relationship between smoking status and angina [43], and the present finding of the study also suggests that the risk of having angina were higher among those who smoked and those who used smokeless tobacco as compared to those who did not smoke or use smokeless tobacco.

### Factors associated with undiagnosed heart disease

Indeed, India is regarded as a particularly relevant LMIC for studying the rising burden of non-communicable diseases (NCDs) such as heart diseases, stroke, cancer, diabetes etc [44]. Although NCDs are rapidly dominating health-care demands in LMICs, and their significance has gained policy acknowledgment in the last decade [30,44–47]. Because of the size of the population and the increasing risk factor profile linked with recent significant economic expansion, India is expected to have more NCD fatalities than any other country during the next decade [48]. The country has profound and entrenched social and economic inequality, with affordable healthcare out of access for huge segments of the population [49]. Meanwhile, the diagnosis of heart failure in long-term care residents is difficult due to co-morbidities, cognitive deficiency, polypharmacy, immobility, and limited access to treatments among older populations [50]. Therefore, the present study also analysed the prevalence and the effect of undiagnosed and uncontrolled heart disease among older adults in India. Besides that, previous study suggests that the onset of valvular heart disease, along with its rise with age, indicates that the burden of such disorders is considerable and growing [51]; however, our findings showed that individuals aged 45–49 years had a greater prevalence of undiagnosed heart disease (68.69%). Besides that, earlier study suggests that valve heart diseases are underdiagnosed in the population, particularly in women, which might be a significant bias given that these illnesses have major physiological and clinical repercussions [51]. In contrast, the present finding of the study found that females are more likely to have undiagnosed heart disease than males.

Socioeconomic status (SES), which is commonly quantified using individual income level, educational attainment level, work status, or neighborhood-level socioeconomic indicators, is a social determinant of health linked to CVD [52]. Previous study discussed that education is commonly related with coronary heart disease risk variables; however, there is still debate over whether this association is independent of seldom assessed confounders [53]. Other putative explaining factors, such as literacy [53,54], time preference [55], and sensation of control [53,56], are less well known. while the present finding showed that individuals with fewer than 10 years and 10 or more years of education were less likely to have undiagnosed heart disease than uneducated individuals. Studies have found that lower individual-level SES are linked to a greater incidence and prevalence of CVD, such as coronary heart disease, heart failure, and stroke [52,57,58]. However, the present finding showed that individuals in the middle and rich wealth quintiles were less likely to have undiagnosed heart disease.

Previous study demonstrated that CVD comorbidities were shown to be favorably linked with diagnosed hypertension, and patients with cardiovascular comorbidities are more likely than those without cardiovascular diseases to seek medical guidance and, as a result, have their hypertension detected earlier [59]. The present finding of the study found that undiagnosed heart disease was less common in hypertension adults than in non-hypertensive individuals. In the older population, diabetes is a significant risk factor for the onset of CVD, and the combined prevalence of diagnosed and undiagnosed prediabetes and diabetes lies between 50 to 80% [60,61]. Our study showed that those individuals with diabetes are significantly less likely to have undiagnosed heart disease compared to the individual with no diabetes.

Furthermore, smoking and usage of smokeless tobacco are one of the world’s most serious public health hazards, and it is a significant risk factor for a variety of chronic illnesses, including cancer, lung diseases, CVDs, and stroke [62,63]. A study conducted in Bangladesh suggests that there was no statistically significant link between smokeless tobacco usage in general and CHD among nonsmokers [64]; but there was a strong association between gul usage and CHD [64]. However, the present finding of the study found that the individuals who smoked or used smokeless tobacco were more likely than nonsmokers to have undiagnosed heart disease. Given the higher prevalence of tobacco-related diseases among those with lower socioeconomic positions, as well as restricted resources for health promotion operations in developing countries, policies encouraging the use of no tobacco at all are justifiable. In India, the strategic focus should be on controlling both smoking and smokeless tobacco usage.

### Factors associated with uncontrolled heart disease

Our study revealed that older adults aged 70 and above years had a higher prevalence of uncontrolled heart diseases compared to middle-aged adults and young-olds age groups, but the age did not show any significant association with uncontrolled heart diseases. The ongoing disparities are a result of historically comparable sex-specific changes in CVD risk variables [65]. Over time, women experienced a greater overall drop in cardiovascular risk factors than males. Interstingly, our study has found that females have significantly lower risk of uncontrolled heart disease compared to men. Household income was found to be substantially and independently related to heart disease [66]. Studies have found that lower individual-level SES are linked to a greater incidence and prevalence of CVD, such as coronary heart disease, heart failure, and stroke [52,57,58]. While our study showed that individuals belonging to the middle and rich income household groups have stronger association with uncontrolled heart disease.

Globally, an estimated 1.28 billion persons aged 30–79 years have hypertension, with the majority (two-thirds) residing in low- and middle-income nations [67]. While in September 2016, WHO and the US Centers for Disease Control and Prevention (CDC) established the Global Hearts Initiative, which includes the HEARTS technical package, to assist countries in increasing cardiovascular disease prevention and control [67]. However, several other health indicators such as hypertension, diabetes and high cholesterol are significantly strongly associated with uncontrolled heart disease, and consistent results have been observed in the previous studies [61,68,69]. Meanwhile, the prevalence of high cholesterol is about 4% only. This is rather low prevalence which may be due to underdiagnosis, especially among low socioeconomic groups.

Furthermore, heart disease is more likely to strike a family member than an unrelated person [70]. Familial hypercholesterolemia, a hereditary illness that causes high cholesterol, can occasionally be detected by the presence of family members who have heart disease at a young age (age 50 or younger). Our study has revealed that individuals with family history of heart diseases have greater risk of uncontrolled heart disease compared to individuals with no family history of heart disease.

### Limitations of the study

The present study has certain drawbacks. Causation could not be established due to the cross-sectional nature of the data used in our research. The present study could only uncover connections; longitudinal analyses may elucidate the causal linkages between socioeconomic and demographic characteristics of heart disease and angina and their diagnosis and control. Greater crucially, the LASI excluded institutionalized older persons, who are likely to have more health issues and a higher burden of diseases than community-dwelling older adults. If some respondents are undiagnosed, the frequency of illnesses may be underestimated, leading to biases, especially if undiagnosed respondents are disproportionately of lower socioeconomic class. The definition of undiagnosed heart disease is too broad. Patients from lower socioeconomic status or illiterate may have been diagnosed and been told to have heart disease but they may not understand and hence will be labelled as undiagnosed heart disease. Likewise, the definition of uncontrolled heart disease is based on if the patients still have symptoms of angina despite the known diagnosis of heart disease. But the symptoms may be caused by non-adherence to the treatment of heart disease. These need to be cautioned while interpreting the current findings.

### Implications for policy, practice and future research

Comprehensive and aggressive control of risk factors is necessary for all patients with stable CAD. For most patients with chronic stable angina, initiating medical therapy alone is suitable and it can form the fundamental aspect of treatment for chronic CAD. When making decisions about patient care, physicians must consider a combination of current evidence-based medicine, the preferences of the patient, and the patient’s expectations regarding their quality of life. As shown in previous studies, implementing a personalized intervention strategy led by community health workers in patients with acute coronary syndrome can enhance adherence to evidence-based medications and healthy lifestyle practices, leading to improvements in clinical risk markers [71]. Thus, incorporating trained community health workers can enhance secondary prevention efforts in coronary artery disease. Also, a comprehensive understanding of the most effective practices learned from previous community-based intervention trials is crucial for designing future population-level cardiovascular prevention interventions.

Further, investigating subclinical endpoints will play a significant role in identifying previously unknown risk factors that could be relevant for primary prevention. Additionally, population-based studies that explore innovative ways to utilize information about established risk factors will be vital for the advancement of strategies in secondary prevention. To advance population-based prevention efforts, it is crucial to prioritize CHD research in specific populations such as women, children, and older adults. Such studies should aim to evaluate the relevance of existing knowledge to these populations and develop interventions tailored specifically to their needs. By addressing the applicability of current knowledge and designing population-specific interventions, we can enhance the effectiveness of CHD prevention strategies.

## Conclusions

The present study provided a comparative prevalence of heart disease and angina and their associations with chronic diseases among middle-aged and older adults in India. The higher prevalence of undiagnosed and uncontrolled heart disease and their risk factors among middle-aged and older Indians manisfest alarming public health concerns and future health demand. Implementation techniques are needed to reduce heart disease and angina risk among older people by focusing on physical activity promotion, early identification (a routine check during medical visits for various diseases may result in more and earlier diagnosis), comprehensive accurate diagnosis of CVDs based on family history is key to promoting CVDs management. It poses a risk if health promotion and awareness initiatives are not effectively developed. Hence, this would leads to the CVD-risk prevention, which is one of the important priorities among India’s sustainable development goals. This study has policy implications for other LMICs in terms of heart disease control. Our findings of high prevalence of undiagnosed and uncontrolled heart disease imply that clinical and public health actions are needed to enhance CVDs’ screening and management. The relationship between undiagnosed heart diseases and a lack of health insurance shows that enhancing primary care coverage, or even simply making patients aware of their insurance entitlement, may help narrow gaps in cardiac care.

## Abbreviations

LASI: Longitudinal Aging Study in India
CVD: Cardiovascular disease
CAD: Coronary artery disease
CHD: Chronic heart disease
AP: Angina pectoris
RAQ: Ross angina questionnaire
